# Maternal autistic traits and anxiety in children with typical development in Chinese families: a moderated mediation model of mothers’ negative emotional expressions and child gender

**DOI:** 10.3389/fpsyg.2024.1264173

**Published:** 2024-02-05

**Authors:** Jiyou Gu, Tiantian Li, Huiqin Dong

**Affiliations:** Faculty of Education, Shandong Normal University, Jinan, China

**Keywords:** maternal autistic traits, children’s anxiety, maternal negative emotional expressions, moderated mediation effect, child gender

## Abstract

**Background:**

Prior studies have focused on the effects of maternal autistic traits on children with autism, but little attention has been paid to the effects of maternal autistic traits on typically developing children, while the mechanisms of the effects are not clear.

**Objective:**

Given that, a moderated mediation model was conducted to examine the association between maternal autistic traits and typically developing children’s anxiety and the underlying mechanisms.

**Methods and results:**

Participants were 648 mother–child dyads in which these children had no autistic siblings. Mothers reported their autistic traits and negative emotional expressions in the family and children’s anxiety. The results indicated that children’s anxiety was predicted by maternal autistic traits. Mediating analysis revealed that mothers’ negative emotional expressions partially mediated the association between their autistic traits and children’s anxiety. The findings also indicated that child gender moderated the relationship between maternal emotional expressions and children’s anxiety. Specifically, anxiety in girls was more strongly predicted by negative emotional expressions from their mothers than in boys.

**Conclusion:**

These results have important theoretical and practical implications for reducing the adverse effect of maternal autistic traits on children’s anxiety, especially for girls. The present study also reveals that maternal negative emotional expression is an important mechanism. Causal conclusions cannot be drawn based on cross-sectional research design, so it is necessary to conduct longitudinal studies in the future.

## 1 Introduction

Autistic traits (ATs) are defined as a collection of personality, behavioral and cognitive characteristics associated with Autistic Spectrum Disorders (ASD) (Sucksmith et al., 2011). It is now commonly acknowledged that ATs exist as a continuum among the general population because of the adoption of the new diagnostic category name, ASD, and the focus on the continuity of ATs in DSM-V (American Psychiatric Association, 2013; Constantino and Charman, 2016). Despite not meeting clinical diagnostic criteria for ASD, individuals with high ATs also exhibit cognitive, emotional, and behavioral characteristics similar to those of individuals with ASD (Gökçen et al., 2014; Bralten et al., 2019).

Current research has concentrated on investigating how parental ATs affect children diagnosed with ASD. Parental ATs were found to relate to both the clinical and subclinical behavioral autistic phenotype of their children (De la Marche et al., 2015; Jones et al., 2017; Riva et al., 2019). However, the effect of parental ATs on their typically developing (TD) children was not well characterized. To our knowledge, only a few studies have been performed to explore this issue to date with inconclusive results regarding child cognitive abilities. For example, researchers used TD infants as participants and found that higher ATs in their parents predicted infants’ atypical visual attention (Jones et al., 2017; Riva et al., 2019); using a longitudinal study, Loncarevic et al. (2021) discovered that parental ATs were associated with TD infants’ communicational difficulties at 24 months; according to Roberta et al. (2021), TD infants’ lower level of statistical learning ability was related to parental ATs.

It is worth noting that although the above research has confirmed an association between parental ATs and cognitive defects in their TD infants, some limitations should be mentioned. First, the aforementioned studies only relate to the cognitive impacts of parental ATs on infants; little is yet known about how parental ATs may predict their child’s emotional problems; second, these studies only examined infants, ignoring the child outcomes at preschool age; third, the underlying mechanisms between parental ATs and child outcomes are still largely unknown.

Emotional problems in preschool children have been of wide interest to researchers. According to Shin et al. (2022), anxiety is one of the most prevalent emotional problems among preschoolers. It is commonly characterized as a condition of constant alertness accompanied by a constricted focus, a feeling of unease, overthinking concern, a perception of vulnerability, a restraint in actions, and an elevated level of sympathetic stimulation (American Psychiatric Association, 2013). The potential long-term detrimental effects of untreated childhood anxiety on several aspects of a child development, including cognitive, social, and emotional functioning, tend to be more severe (Barlow, 2002; Merikangas et al., 2009; Pereira et al., 2014; Beato et al., 2018). Therefore, determining the correlates of preschool children’s anxiety is extremely important in order to lessen their anxiety and encourage healthy adaption and growth.

Both theory and research have shown that maternal factors are a significant influence on children’s anxiety. Based on Ecological System Theory, the family serves as the primary and influential microsystem in a child’s development (Bronfenbrenner, 1979), within which direct and indirect effects of mothers on children’s anxiety have been confirmed by numerous studies, such as mothers’ emotion (Möller et al., 2015; Ma et al., 2016; Zhao et al., 2018; Măirean et al., 2022), parenting practices (Ahmadzadeh et al., 2019) and mother–child relationships (Casline et al., 2021). In addition, maternal personality has also been the focus of relevant research. For instance, most studies discovered that higher level of maternal neuroticism (Prinzie et al., 2005) and lower level of agreeableness and conscientiousness (Oliver et al., 2009; Xing et al., 2018) have been associated with higher levels of anxiety in their offspring (Crawford et al., 2011; Puff and Renk, 2016). However, all of the above studies were based on the Five Factor Model (McCrae and Costa, 1987), ignoring to examine maternal ATs, which is the sixth factor of personality (Wakabayashi et al., 2006).

It has been proposed that negative aspects of maternal personality, such as poorer social and communication abilities and insensitivity to children, may be significant contributors to children’s anxiety (Crawford et al., 2011). Given that social and communication skills are the fundamental components of ATs (Gökçen et al., 2014; Yang et al., 2018), and that maternal social and communication skills decreases as the level of ATs increase, we proposed hypothesis H1: Maternal ATs positively predict children’s anxiety.

Individuals with high levels of ATs express more symptoms/higher levels of depression and anxiety. Albantakis et al. (2020), for instance, discovered that ATs were predictive of symptoms of social anxiety disorder and depression; according to Culpin et al. (2018), adolescents with high levels of ATs generally had high levels of depression; Cai et al. (2019) suggested that people with ASD may be more prone to aggression, anxiety, and sadness than TD controls. Consequently, it is probable that the level of maternal ATs predicts their negative emotional expressions in the family.

It is well-known that negative emotions in mothers are directly linked to anxiety in children. On one hand, according to the model of emotional socialization and social learning theory, mother’s emotional reaction to situations serves as an example for children to imitate (Bandura, 1977; Eisenberg et al., 1998).

As a result, when mothers display negative emotions more frequently, children are more likely to experience them, such as anxiety and depression. On the other hand, studies about the intergenerational transmission of anxiety and depression have indicated that when mothers express their children anxiety and despair more, their children are more likely to experience similar feelings as well. Based on the above analysis, we proposed hypothesis H2: Mothers’ negative emotional expressions play a mediating role between their ATs and children’s anxiety.

**Figure 1:**
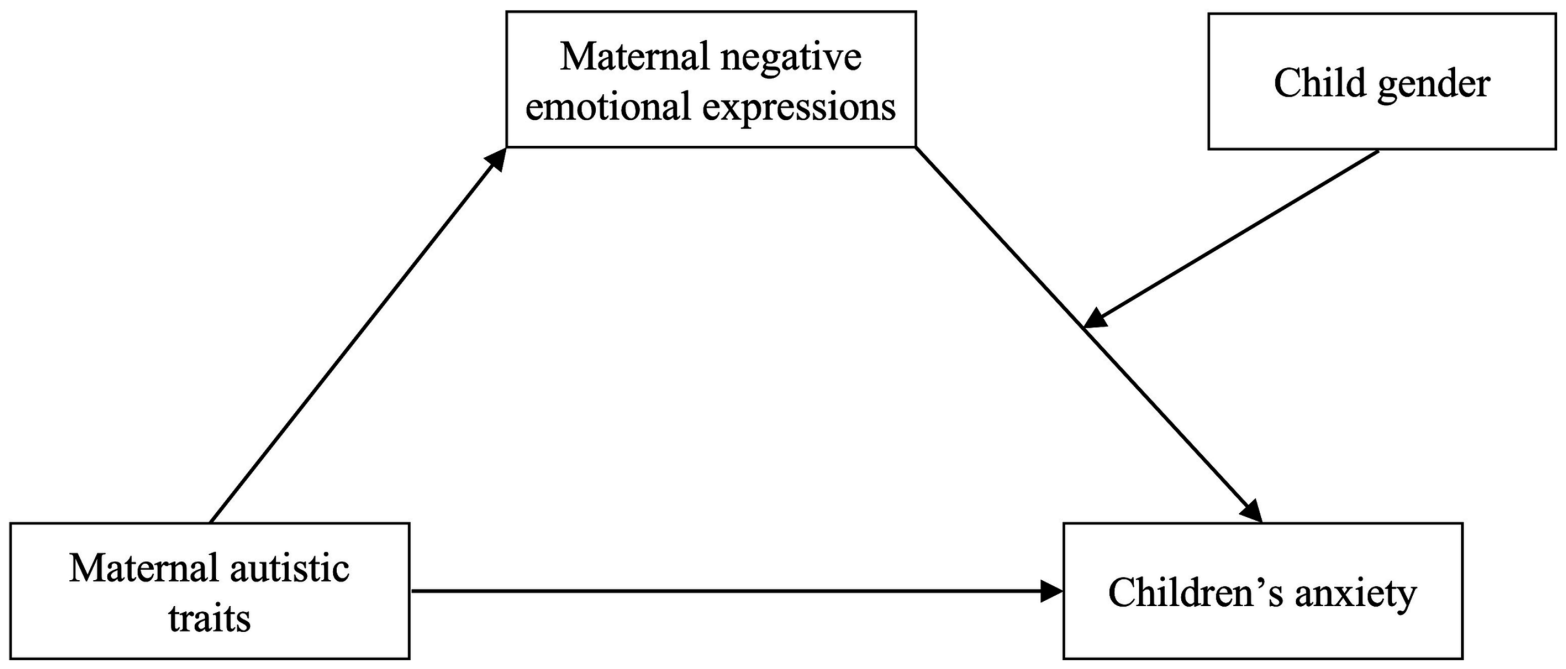
Conceptual framework.

It is worth noting that boys and girls could be differently impacted by their mothers’ negative emotional expressions. The Gendered Social Learning Model suggested that children may tend to consider same-gender parents to be role models (Deater-Deckard and Dodge, 1997). Thus, girls are consequently more likely to acquire their mothers’ emotional reactions. In addition, girls encode negative information in greater detail, absorb it more thoroughly, and are more prone to acquire an attention bias toward negative information, such as mothers’ negative emotion, than boys (Perchtold et al., 2019). As a result, girls may be more receptive to their mothers’ negative emotional responses because they are more sensitive and emotionally connected to their mothers than boys (Tottenham et al., 2011). That means, girls experience more anxiety compared to boys. Accordingly, the present study proposes the hypothesis H3: Gender plays a moderating role in the relationship between children’s anxiety and mothers’ negative emotional expressions. In summary, this research presents a hypothesis model (Figure 1) and empirically investigates the aforementioned hypothesis, providing mothers with high ATs in Chinese families theoretical direction and an empirical foundation for scientific prevention and successful management of the degree of anxiety in their children.

## 2 Methods

### 2.1 Procedure

The children in this study were from six kindergartens in Jinan and Dezhou, the province of Shandong in eastern China. The study was carried out over the course of 3 months, from June to August 2022. After giving mothers through the kindergartens informed consent forms and inviting them to attend lectures on family education and child development, the mothers filled out questionnaires. Each mother received a gift worth ¥30 in return for completing the questionnaire. All study procedures were authorized by Shandong Normal University’s Institutional Review Board.

### 2.2 Participants

Using the cluster sampling method, the original sample for this study consisted of 665 mothers and their TD children (4–6 years old). After recall, invalid questionnaires such as too many omissions, random responses and regular responses were excluded, and the final sample included 648 mother–child dyads with a questionnaire validity rate of 97.44%. Mothers’ ages ranged from 25 to 49 years (*M* = 36.53; SD = 7.22). According to data on socioeconomic status, 61.8% of mothers had a college degree or higher, while just 17.1% of mothers had completed high school. Regarding employment, 54.11% of the mothers were employed in working-class jobs (e.g., factory workers), while 45.89% of the mothers held a professional, technical, or managerial position (e.g., teachers). Mothers in the present study do not have ASD children. All the children were from public kindergartens. The mean age of the children (352 boys and 280 girls) was 5.03, SD = 0.82. 203 were 4-year-olds, 208 were 5-year-olds, and 221 were 6-year-olds. 28.3% of the children were the only child.

### 2.3 Measures

#### 2.3.1 Maternal ATs

The Autism Spectrum Quotient (AQ) questionnaire, a self-administered tool for assessing ATs in adults with normal intelligence, was used to test maternal ATs (Zhang et al., 2016). The five components of the measure include verbal communication, social skills, attentional shifts, attention to detail, and creativity. Each dimension is scored on a 4-point scale, and mothers report the results. The AQ score is calculated as the sum of the scores for each dimension; higher scores denote higher levels of ATs. The scale has high reliability and validity in existing studies (Meng et al., 2021). In the present study, the Cronbach’s α coefficient of the total scale was 0.785.

#### 2.3.2 Mothers’ negative emotional expressions

The Chinese version of the Self-Expressiveness in the Family Questionnaire was used to test mothers’ negative emotional expressions (Liang et al., 2012). The scale (40 items) consists positive (e.g., “I feel angry at my family’s carelessness”) and negative (e.g., “I feel angry at my family’s carelessness”) emotional expressions, which were scored on a 9-point Likert scale. Only the negative-emotional expression dimension was employed in this research, and the Cronbach’s α coefficient was 0.896.

#### 2.3.3 Preschool children’s anxiety

Mothers’ reports of their children’s anxiety were evaluated using the Chinese version of the Spence Children’s Anxiety Scale for Parents (SCAS-P; Spence, 1998; Wang et al., 2016). Of the 44 items on the scale, 6 are positively worded filter items that cannot be scored. Children’s anxiety was obtained on a three-point scale (from 0 “never” to 3 “always”) by their mothers. The preschooler’s anxiety score on this scale is calculated as the average of all item scores; greater scores correspond to higher levels of anxiety. The scale has high reliability and validity in existing studies (Ding et al., 2022). The Cronbach’s α coefficient for the total scale in this study was 0.893.

### 2.4 Data analysis

This study used SPSS 24.0 and its PROCESS software package for preliminary analysis of the data, which was used to check the completeness of the data and to calculate correlations. Preliminary analysis of the data was performed, which included descriptive statistics and correlation analysis of all data. Mediated effects and tests of mediated effects with moderation were analyzed using the SPSS process plug-in. First, the mediation model was tested to confirm the mechanism of influence of mothers’ negative emotion expressions between mothers’ ATs and children’s anxiety. We calculated the 95% CI based on 5,000 bootstrap samples. Second, the mediating effect with moderation was tested. If the regression coefficients of the interaction term between child gender and mothers’ negative emotion expressions were significant, the mediating model with child gender as the moderating variable was valid. Child gender was assigned separate values, boys = 1 and girls = 2.

## 3 Results

### 3.1 Preliminary analysis

Table 1 shows the relationship between the key factors and demographic variables of this study. There were significant positive correlations between mothers’ levels of ATs and their expressions of negative emotion, mothers’ ATs and children’s anxiety, and mothers’ expressions of negative emotion and children’s anxiety, as shown in Table 1. A multivariate analysis of variance (MANOVA) of 2 (child gender: boy and girl) × 3(child age: 4-, 5-, and 6-years old) was conducted for children’s anxiety. The results revealed that the main effect of child gender, the main effect of child age, and the interaction effect of child gender and age (*ps* > 0.05) for children’s anxiety were not significant.

**Table 1:** Descriptives and correlations between study variables.

Variables	1	2	3	4	5
1. Child gender (1 = boy, 2 = girl)	–				
2. Child age	0.008	–			
3. Mothers’ ATs	−0.044	0.035	–		
4. Mothers’ negative emotional expression	0.031	−0.014	0.146^***^	-	
5. Children’s anxiety	−0.003	−0.009	0.325^***^	0.254^***^	–
M	0.557	4.55	2.330	4.100	2.068
SD	0.497	1.116	0.198	1.335	0.494

*p < 0.05; **p < 0.01; ***p < 0.001.

### 3.2 Mediation analyses

The PROCESS macro procedure proposed by Hayes (2012) was used to test the hypothesized model. A simple mediation model was first tested on the data using Model 4 to explore the mediating effect of mothers’ negative emotion expressions between mothers’ ATs and children’s anxiety. Table 2 shows that mothers’ ATs are directedly and positively predictive of children’s anxiety (*β* = 0.275, *p*<0. 001) and are positively associated with mothers’ negative emotional expressions (*β* = 0.166, *p*<0. 001), which is positively related to children’s anxiety (*β* = 0.227, *p*<0. 001).We calculated the 95% CI based on 5,000 bootstrap samples. It confirmed the significant indirect effects of mothers’ negative emotional expressions in the relationship between their ATs and children’s anxiety [*β* = 0.038,95% CI (0.018, 0.061)]. These results indicated that mothers’ negative emotional expressions partially mediates the relationship between their ATs and children’s anxiety.

**Table 2:** Testing the mediation effect of mothers’ negative emotional expression in the association between mothers’ ATs and children’s anxiety.

Predictor	Model 1 (Mothers’ negative emotional expression)			Model 2 (Children’s anxiety)		
	*β*	SE	t	*β*	SE	*t*
Mothers’ ATs	0.167	0.264	4.228^***^	0.275	0.093	7.422^***^
Mothers’ negative emotional expression				0.226	0.014	6.139^***^
Child age	0.022	0.047	0.552	−0.017	0.016	−0.459
Child gender	0.036	0.104	0.926	0.005	0.036	0.131
Mothers’ career	0.041	0.061	1.906	−0.038	0.021	−0.798
Mothers’ educational level	0.098	0.076	1.906	−0.091	0.027	−1.901
*R^2^*	0.039			0.163		
*F*	5.142^***^			20.834^***^		

^***^p< 0. 001; ^**^p < 0. 01; ^*^ *p* < 0. 05.

### 3.3 Moderated mediation analysis

**Figure 2:**
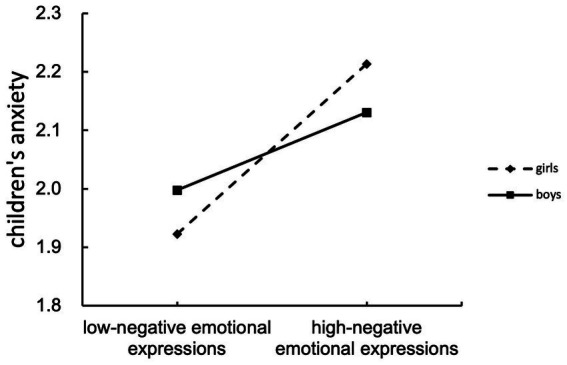
Relation between mothers’ negative emotional expressions and children’s anxiety as moderated by child gender.

Using Model 14 of PROCESS macro (Hayes, 2012), we conducted the moderated mediation analysis. As shown in Table 3, the interaction of mothers’ negative emotional expressions and child gender has a significant effect on children’s anxiety (*β* = 0.062, *p*<0.001). Furthermore, a simple slope analysis was conducted and we plotted the effect of mothers’ negative emotional expressions on children’s anxiety separately for boys and girls (Figure 2). The simple slope test showed that mothers’ negative emotional expressions were a stronger predictor of anxiety in girls (*β* = 0.111, *p*<0.001) compared to boys (*β* = 0.049, *p*<0.05). The conditional indirect effect of mothers’ ATs on children’s anxiety via mothers’ negative emotional expressions was also calculated, as a function of child gender.

**Table 3:** Testing the moderated mediation effect of gender on the relationship between mothers’ negative emotional expression on children’s anxiety.

Predictor	Model 1 (Mothers’ negative emotional expression)				Model 2 (Children’s anxiety)		
	*β*	SE	*t*		*β*	SE	t
Mothers’ ATs	1.107	0.264	4.194***		0.677	0.092	7.337***
Mothers’ negative emotional expression					0.083	0.014	6.125***
Child gender					0.005	0.036	0.139
Mothers’ negative emotional expression × child gender					0.062	0.027	2.299*
Child age	0.025	0.047	0.547		−0.007	0.016	−0.408
Mothers’ career	0.045	0.060	0.741		−0.017	0.021	−0.811
Mothers’ educational level	0.149	0.076	1.967		−0.047	0.026	−1.792
*R^2^*	0.037				0.170		
*F*	6.215***				18.731***		

^***^p < 0. 001; ^**^p < 0. 01; ^*^p < 0. 05.

In addition, the bootstrapping results showed that the conditional indirect effect of mothers’ ATs on children’s anxiety via mothers’ negative emotional expressions was significant by child gender, with the effect being 0.062, SE = 0.030, 95% CI [0.005, 0.120]. Specifically, the conditional indirect effect in girls [*β* = 0.123, 95% CI (0.058, 0.206)] was stronger than that in boys [*β* = 0.054, 95% CI (0.007, 0.112)].

## 4 Discussion

Expanding on prior findings, the present research examined the association between maternal ATs and children’s anxiety. Additionally, it explored the mediating role of negative emotional expressions exhibited by mothers, as well as the potential moderating effect of child gender. Our results showed a relationship between maternal ATs and children’s anxiety, which may be partially attributed to mothers’ negative emotions. In addition, compared to boys, girls experienced higher levels of anxiety when their moms exhibited more negative emotions toward them. These results will be discussed as followed.

### 4.1 The relationship between maternal ATs and children’s anxiety

This study examined, for the first time among the general population, the direct predictive impact of maternal ATs on children’s anxiety. This might be explained by the cognitive similarities between individuals with high level of ATs and those with ASD. Studies of individuals from the general population who scored highly on ATs have shown a pattern of cognitive characteristics that is similar to but less severe than that commonly seen in ASD. For example, Saure and colleagues found the improved cognitive rigidity among individuals with high ATs (Saure et al., 2022). That means, as levels of maternal ATs increased, these mothers came to develop negative interpretations when confronted with ambiguity and uncertainty in their surroundings in the present study. Thus, these mothers might warn their children about potentially dangerous stimuli and circumstances or tell them that the surroundings are dangerous. Consequently, children’s interpretations of ambiguous circumstances might be adversely biased when parents convey to children their negative impressions or assessments of those events, such as by overemphasizing risk factors in the surroundings and by vocally conveying dangerous information regarding unclear stimuli and circumstances. Additionally, due to observational learning, children who repeatedly see their mothers’ cognitive rigidity in reaction to new and unclear environmental stimuli are more likely to experience anxiety and terror of these stimuli (Eley et al., 2015; Aktar et al., 2017). As a result, children’s level of anxiety may increase.

### 4.2 Mediating effect of mothers’ negative emotional expressions in the family

The second hypothesis of this study was validated by showing that mothers’ negative emotion expressions played a mediating role between their ATs and children’s anxiety. Consistent with existing research, the current investigation discovered a correlation between increased expressions of negative emotions and higher levels of maternal ATs. This may be explained by the similarity of emotional characteristics between high levels of ATs and ASD. As reviewed above, mood problems and ASD frequently coexist (White et al., 2018). The majority of studies have discovered that individuals with ASD frequently feel and display unpleasant emotions including anxiety and depression (Joshi, 2017; Taylor et al., 2022). Additionally, prior research has indicated that people with high levels of ATs exhibit emotional regulation deficiencies comparable to those in ASD, with a propensity to be unable to effectively moderate their negative emotions (Cai et al., 2019; Zhao et al., 2020).

Moreover, the present study found that maternal negative emotion expressions significantly and positively predicted children’s anxiety, which is consistent with our expectations. Research has shown that mothers’ emotional expression and regulation significantly affect the physical and mental health of family members (Morris et al., 2017; Risley et al., 2021). Mothers’ sadness and anxiety, in particular, have been shown to have intergenerational transmission consequences. On one hand, the intergenerational transmission model of depression and anxiety (Goodman, 2020; Åhlén et al., 2022) suggests that mothers with higher levels of depression and anxiety may cause themselves to neglect their children’s needs, especially by being less sensitive to their children’s emotional responses and less feedback and supportive, while generating more resentment, derogation, and blame for their children, which leads to higher levels of anxiety in their children. On the other hand, based on Social Learning Theory (Bandura, 1977; Fu et al., 2022), anxiety is acquired by observing mothers’ fearful, apprehensive reactions to environmental stimuli. For example, if children witness their moms reacting nervously to ambiguous or somewhat threatening stimuli, they will react anxiously when they are in the situation themselves. In addition, the model of emotional socialization suggests that parental emotional expression is a reference paradigm for children (Denham, 1998; Denham and Burton, 2003; Fu et al., 2022). For instance, if mothers express more negative emotions, children learn this pattern of expressions and tend to express negative emotions as well. Consequently, more expressions of negative emotions by mothers tend to create a depressing and negative emotional climate in families (Fredrick et al., 2019; Khoshroo and Seyed Mousavi, 2022), which in turn increases children’s negative emotional experiences and increases the incidence of internalizing problems (Hu et al., 2017), leading to increased levels of anxiety in children. These findings imply that mothers’ negative emotional expressions may be one mechanism by which maternal ATs predict children’s anxiety.

### 4.3 Moderating effect of child gender

Consistent with the hypothesis, the present study discovered the moderating role of child gender. Specifically, girls experience more anxiety when their mothers expressed more negative emotions toward them compared to boys. There are three possible explanations as to the finding of the moderated effect of child gender. First, guided by the gender-specific social learning model, children view same-gender parents as role models and imitate their behavior patterns, so girls express anxiety with their mothers as targets of social learning. Second, girls are more sensitive than boys (Milfont and Sibley, 2016) and have stronger internal tension and anxiety when confronted with mothers’ anxiety. Finally, mothers may have more emotional interactions and connections with girls in Chinses family (Ming et al., 2020), which lead girls to be more vulnerable than boys to high levels of mothers’ negative emotions, resulting in experiencing more anxiety.

### 4.4 Limitations and future direction

It is important to acknowledge that this study has several limitations, even if it offers a deeper understanding of the impact of maternal ATs on children’s anxiety in Chinese families for the first time.

First, this study used maternal ATs as an independent variable and did not consider whether and how paternal ATs affect children’s emotional problems. It has been suggested that there are gender differences in ATs, as evidenced by generally higher levels of ATs in males than in females (Baron-Cohen et al., 2001; Pisula et al., 2013). Therefore, future studies could select fathers as subjects to compare with the results of this study to fully explore the relationship between the level of parental ATs and children’s emotional problems. Second, it is challenging to make conclusions from the cross-sectional data on the associations between mothers’ ATs, children’s anxiety, and mothers’ negative emotional expression. To clearly establishing causal and temporal conclusions, longitudinal investigations are required in the future. Third, because preschoolers were too young to complete the questionnaires, this study only used mother-report data, which could artificially inflate some of the associations across variables due to shared method variance. To solve this issue, more research evaluating children’s anxiety using a variety of techniques will be required. Finally, the Family Self-Expression Inventory was used in the present study, which includes a variety of negative emotions but not concentrating on a certain feeling such as anxiety and depression. Future studies could select maternal anxiety and depression as mediating variables, respectively, to further explore the mechanisms by which maternal ATs affect children’s anxiety in detail. Moreover, it has been demonstrated that, like ASD, individuals with more ATs are insensitive to emotional information from others, have difficulty recognizing emotions (Cai et al., 2019), and lack certain empathic abilities (Vargas et al., 2005), which prevent mothers with high levels of ATs from detecting their children’s anxiety in a timely manner and from helping their children relieve their anxiety through acceptance, empathy, and understanding, thus leading to increased anxiety in their children. Future research needs to specifically analyze the mediating pathways through which mothers’ ATs affect their children’s anxiety in terms of emotion recognition and empathy.

### 4.5 Implications

Despite these drawbacks, the present study offers some insightful data and significant practical applications. First, this is the first study, to our knowledge, to explore the impact of ATs in general population in Chinese families. The findings of this study will expand the scope of research in the field of ATs and provide a research base for further exploration the impact of adult ASD individuals on family members, especially for their children. Second, the present study examined the association between maternal ATs and children’s anxiety in the general population. The results of this investigation provide a significant addition and expansion to earlier analyses of the mechanisms underlying children’s anxiety in family systems. Finally, the present study found that maternal negative emotional expressions mediated their ATs and children’s anxiety and the moderated effect of child gender. This conclusion emphasizes, especially for girls, that effective preventative measures aimed at lowering the level of children’s anxiety, particularly for mothers in the family who reported more ATs, should target not just the children themselves (e.g., children’s negative interpretation bias), dyadic factors (e.g., parent–child relationship), but also the function of maternal personality traits.

## Data availability statement

The raw data supporting the conclusions of this article will be made available by the authors, without undue reservation.

## Ethics statement

The Institutional Review Board of Shandong Normal University approved all study procedures. The patients/participants legal guardian/next of kin provided written informed consent to participate in this study.

## Author contributions

JG: Writing – original draft. TL: Writing – original draft, Writing – review & editing. HD: Writing – review & editing.
